# Effectiveness of Sensors-Based Augmented Feedback in Ergonomics to Reduce Adverse Biomechanical Exposure in Work-Related Manual Handling—A Rapid Review of the Evidence

**DOI:** 10.3390/s24216977

**Published:** 2024-10-30

**Authors:** Carl M. Lind

**Affiliations:** Unit of Occupational Medicine, Institute of Environmental Medicine, Karolinska Institutet, 171 77 Stockholm, Sweden; carl.lind@ki.se

**Keywords:** augmented feedback, biofeedback, intervention, work postures, muscle activation, musculoskeletal disorders, musculoskeletal pain, wearables, work technique, ergonomics

## Abstract

Manual handling is a major risk factor for work-related musculoskeletal disorders and one of the leading causes of disability-adjusted life years globally, necessitating multifaceted risk reduction measures. One potential intervention for manual handling tasks is work technique training assisted by augmented feedback on biomechanical exposures. However, there is a research gap regarding its effectiveness specifically for manual handling tasks in both real work environments and controlled settings, as well as its ability to induce retained reductions in biomechanical exposure. The gap was investigated using a rapid review comprising a literature search using two databases and 11 reviews/overviews to identify studies from the past 20 years, up to studies published by 1 June 2024. Sixteen studies were identified, with 14 of them being of high or moderate methodological quality and were included. Three studies were conducted in real work environments and eleven in controlled settings. Most studies (n = 9) used auditory feedback, followed by vibration feedback (n = 6). In real work environments, the evidence for the effectiveness of sensor-based augmented feedback in reducing biomechanical exposure during administration was considered to be inconsistent and very limited directly after administration. For longer periods after administration, ranging from one week to more than six months, there is currently no evidence demonstrating the effectiveness of the feedback. In controlled settings, there was strong evidence for its effectiveness during and immediately after administration, and limited evidence for effectiveness up to six months post-administration when considering the tasks included in the training. Future research needs are discussed.

## 1. Introduction

### 1.1. Work-Related Disorders and Work Technique Training in Manual Handling

Work-related diseases and disorders remain a major global health concern, affecting an estimated 1.7 billion people worldwide who experience ill musculoskeletal conditions [1]. Musculoskeletal disorders (MSDs), such as low back pain and neck pain, alone account for an estimated 95 million disability-adjusted life years globally [2]. Work-related musculoskeletal disorders (WMSDs) and occupational accidents and diseases are estimated to cost 3.9% of the global gross domestic product and 3.3% within the European Union [3] In addition, impairing work capacity and increasing the risk of short- and long-term absenteeism, WMSDs can lead to premature exit from the labor market [4,5,6]. The etiology of WMSDs is multifactorial, involving both physical and psychosocial risk factors [7,8,9]. Major physical risk factors frequently occurring in workplaces include repetitive and heavy manual handling [7,10,11,12,13,14,15,16,17,18], demanding postures and movements [7,10,12,18,19,20,21,22], and hand-arm and whole-body vibrations [7,10,23,24].

Preventive management of hazardous manual handling encompasses screening and risk assessment of exposures, as well as implementing risk-reducing measures to eliminate or mitigate the identified hazards [25,26,27,28,29,30]. The use of mechanical lifting equipment is commonly employed to reduce WMSD risks related to manual handling tasks [31,32,33,34], as well as work technique training aimed at minimizing adverse postures or movements [35,36,37,38]. Work technique training is mandatory in the European Union to mitigate hazardous work-related manual handling [39]. The training often aims to minimize the occurrence of stressful postures, such as a flexed or rotated trunk during force exertion, as these postures increase the risk of WMSDs [33,40,41,42,43].

Traditional training to improve work technique typically comprises theoretical education on safe work techniques and brief practical training sessions designed to incorporate the technique. While this approach may be effective in certain contexts, recent systematic reviews have concluded that such training has typically little to no clinically relevant impact on reducing WMSDs [38,44,45,46]. For greater effectiveness, it has been recommended that training be conducted over a longer period to facilitate motor learning and include more realistic manual handling tasks instead of a limited subset of simplified tasks [38]. Such an extension of the training period may incur high costs, including a prolonged need for an instructor, and may often be considered infeasible. To address this issue, one potential solution is to complement traditional training with the use of sensor-based biofeedback technologies [47]. These technologies provide various systems for exposure- and risk assessment that can be utilized in work contexts, and worker training is assisted by augmented feedback through visual, auditory, or tactile cues [47,48,49,50].

### 1.2. Current Research on the Effectiveness of Sensor-Based Augmented Feedback Training

Sensor-based biofeedback has been used for several decades in ergonomics research to reduce adverse exposure in work settings, with a considerable proportion of studies focusing on computer tasks (e.g., [51,52,53,54,55,56,57,58]). Typically, electronic devices used in previous studies for collecting kinetic data included triaxial accelerometers, inertial measurement units (IMUs), twin-axis electrical goniometers, and video-based motion capture systems with reflective markers, as well as surface electromyography (sEMG) to collect data on muscle activity [47]. Augmented feedback can be used to provide delayed (terminal) or real-time (concurrent) cues to inform workers about adverse postures, guiding them to adopt less harmful postural behaviors. For example, this may involve reducing trunk flexion and rotation, which place the trunk in vulnerable positions [59,60,61,62]. Such guidance can help improve posture and movement patterns, potentially reducing the risk of injuries and musculoskeletal disorders. Over the past 10–15 years, several studies have been published, applying wearable sensor-based systems to reduce adverse postures and movements in work-related tasks. The application of wearable sensor technologies, including augmented feedback, to mitigate work-related musculoskeletal disorders (WMSDs) has been reviewed in several recent studies [47,48,49,50,63,64,65,66,67,68,69]. However, most of these reviews lack an assessment of the methodological quality of the studies they reviewed [47,48,49,50,63,64,68] and have not evaluated the strength of the evidence for the effectiveness of augmented feedback [47,48,49,50,63,64,65,68].

However, the reviews by Lee et al. [66] and Lind [67] included both assessments of methodological quality and grading of the evidence for the effectiveness of sensor-based augmented feedback in reducing adverse exposures related to WMSDs. Additionally, Frasie et al. [66] assessed the methodological quality and the strength of the evidence for extrinsic feedback for both MSD prevention and rehabilitation and had a broader scope that included feedback from instructors such as therapists, not limited to only sensor-based feedback. None of these three reviews exclusively targeted manual handling tasks. Instead, assessment of the strength of evidence for the effectiveness of augmented feedback was based on a broad range of diverse tasks, including sedentary computer entry tasks, odontology tasks, and manual handling tasks such as order picking and patient transfer. This mixture of tasks ranges from sedentary activities with lower force exertions and more static postures to tasks with more dynamic movements and moderate- to high physical exertion demands. Given the distinct nature of these different task types, there is a need to evaluate the effectiveness of augmented feedback separately.

The study by Lee et al. [66] did not differentiate the effectiveness of interventions in controlled settings from those in real work environments, nor did it assess the strength of evidence over different time frames to differentiate direct effects from retained effects. Lind [67] assessed the strength of evidence separately for studies conducted in controlled settings versus real work environments, as well as the temporal aspects (short, moderate, and long-term); the study was however limited to wearable motion capture systems. Consequently, studies targeting a reduction in adverse exposure in manual handling using EMG or non-ambulatory sensor-based technologies were omitted as they did not fulfill the inclusion criteria (e.g., [70,71]). Similarly, several studies evaluating the effectiveness of sensor-based feedback were not included in the reviews by Lee et al. [66] (e.g., [72,73,74]) and Frasie et al. [66] (e.g., [73,75,76,77]).

Based on the current literature, there is a gap in the research literature regarding the evidence for the effectiveness of sensor-based augmented feedback to mitigate adverse biomechanical exposure in manual handling tasks. There is also a need to differentiate the evidence for the effectiveness in real work environments versus controlled settings (i.e., laboratory settings) and to distinguish their short-term and long-term effects.

### 1.3. Aim

This rapid review aims to fill the identified research gap by evaluating the evidence for the effectiveness of sensor-based augmented feedback in reducing biomechanical exposure of the upper body in work-related manual handling tasks. The evaluation of the evidence considers temporal aspects of feedback deployment (direct, short-term, mid-term, and long-term effects) as well as the specific settings for its use (real work environments versus controlled settings).

## 2. Materials and Methods

A rapid review design was applied, following the Cochrane Rapid Reviews Methods Group guidelines for rapid reviews [78] and structured using the PRISMA 2020 guidelines [79].

### 2.1. Eligibility Criteria

To be eligible, the source had to be a peer-reviewed journal article written in English, presenting an evaluation of augmented feedback from data recorded by sensors aimed at reducing biomechanical exposure in work-related manual handling tasks in an adult population. Studies focusing on rehabilitation and sports were excluded (Table 1).

### 2.2. Search Strategy

**Figure 1:**
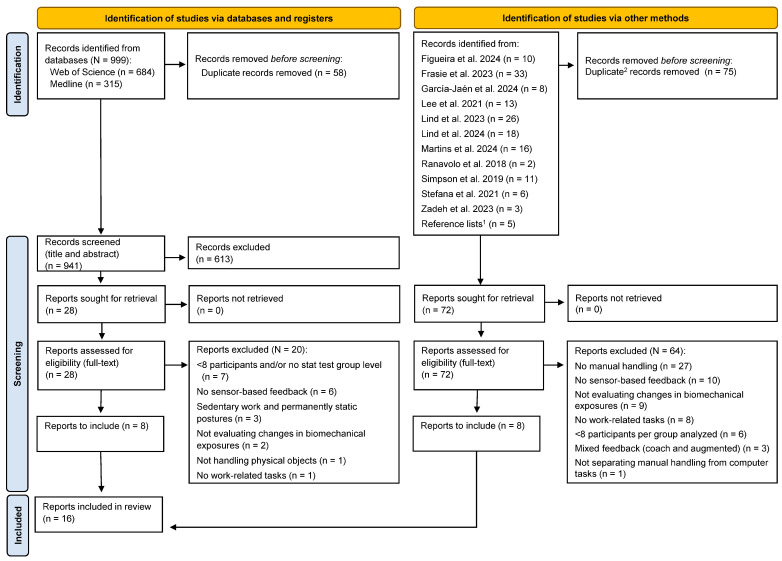
PRISMA 2020 flow diagram of the inclusion process [47,48,49,50,63,64,65,66,67,68,69]. Notes: ^1^ additional reference was retrieved from the reports included in the database search; ^2^ duplicate records were included from the database search and those identified from the 11 reviews and overviews.

To identify relevant literature, 11 recent reviews were used as the basis, along with a systematic electronic literature search to identify newly published literature (Figure 1). The reviews covered different periods and scopes. For example, the review by Frasie et al. [66] covered studies from 1986 to 2 August 2022, whereas Lee et al. [69] covered studies from 2005 to 15 July 2021. To identify literature published after 2020, a systematic electronic literature search was conducted using the databases by Medline and Web of Science. The search period was 1 January 2020 to 9 June 2024 (see Appendix A, Table A1 and Table A2). Additionally, the reference lists of included articles and the author’s personal libraries were used to retrieve additional records. Duplicate records were removed using the function *Remove Duplicates* in Microsoft 365 Excel (version 2304, Microsoft Corporation, Redmond, WA, USA), followed by a manual check to identify and exclude any potential remaining duplicates.

### 2.3. Study Selection

The eligibility criteria were applied to the identified articles by one reviewer (C.M.L.) based on their titles and abstracts (Figure 1). If eligibility could not be determined from the titles and abstracts, the full text was then assessed.

### 2.4. Methodological Quality Assessment

To assess the methodological quality, the tools to assess controlled intervention studies and observational cohort and cross-sectional studies by the National Heart, Lung, and Blood Institute [80] were used (see Appendix B, Table A3 and Table A4). Recognizing the potential variance in quality scores arising from different methodological assessment tools, it was predetermined to restrict the selection to these two tools.

One reviewer (C.M.L.) assessed the methodological quality by assessing the pre-defined criteria as follows: fulfilled, not fulfilled, not applicable, or cannot be assessed due to insufficient reporting (i.e., *not reported*). Fulfilled criteria were assigned one point, and criteria that were either not fulfilled or not reported were assigned zero points. For criteria judged as not applicable, a deduction of the maximum score was made. Subsequently, the score was utilized to classify the methodological quality as follows: high quality (≥75% of the maximum score), moderate quality (50–74% of the maximum score), and low quality (<50% of the maximum score).

### 2.5. Strength of Evidence Assessment

The strength of evidence (see Table 2) was assessed by one reviewer (C.M.L.) utilizing a seven-category scale ranging from *no evidence* to *strong evidence* adapted from Lee et al. [69] and Lind [67].

### 2.6. Data Extraction

The following information of the included studies were extracted by one reviewer (C.M.L.):

- Targeted outcome
- Study design (including the use of a control group)
- Setting and tasks performed
- Participants’ characteristics (sex, age, and eligibility)
- Feedback evaluation and duration of the retention tests (see Table 3)
- Feedback characteristics (type and modality, targeted body region, and feedback trig-ger)
- Equipment for collecting and analyzing exposure data (including if it is ambulatory).

Additionally, the force demands and task complexity of the tasks performed by the participants were subjectively assessed based on the information reported and general experience with similar tasks.

## 3. Results

After removing duplicates, a total of 1013 unique records were identified from the literature search (Figure 1) and underwent screening based on their titles and abstracts, with 100 undergoing full-text assessment. Upon application of the eligibility criteria, 16 peer-reviewed articles, each comprising one relevant study, were deemed fulfilling the assessment of inclusion.

### 3.1. Quality Assessment

The assessment of the methodological quality of the 16 identified studies is shown in Table 4 and Table 5. For two studies, the methodological quality was assessed using both quality assessment tools, resulting in full agreement of the quality ratings between the tools. Among the 16 identified studies, eight (50%) were classified as having high quality, while six (38%) were classified as having moderate quality. Furthermore, two studies (13%) were classified as having low methodological quality and consequently were excluded. Hence, 14 studies were included in the synthesis.

The methodological quality criteria with the lowest fulfillment rates of the 16 initial studies were:

- Not reporting the participation rate of eligible persons in the identified pool of eligible persons (n = 15) (Criterion 3).
- Not blinding (or reporting blinding) of assessors to the participants’ group allocation (n = 15) (Criterion 12).
- No clear justification of the sample size to detect a difference in the outcome with at least 80% power (n = 13) (Criterion 5).

### 3.2. Study Design, Methodology, and Instruments

#### 3.2.1. Study Design, Settings, Tasks and Participants

As shown in Table 6, a cross-sectional design was the most common study design, used in 11 studies, while two studies used a combination of cross-sectional and longitudinal designs. Only one study employed a cluster randomized control trial design (RCT) [74]. A control group was used in half of the studies, while the rest predominantly compared the effect of the feedback to the baseline. All studies, except for Lind et al. [86], targeted a reduction in biomechanical exposure of the spine, including reductions in spine flexion or inclination, moment, or lumbosacral compression force. Besides the spine, three studies [73,76,86] targeted a reduction in upper arm elevation exposure. The majority of studies (n = 11) were conducted in controlled settings, while three studies were carried out in real work environments where the subjects performed regular work tasks.

As shown in Table 7, the most common task evaluated was related to handling box-type items, which was explicitly reported to be used in eight studies (57%). Health care and care tasks were performed in four studies (29%); two studies exclusively targeted patient transfer, while the other two included various activities performed in real work environments. The assumed force demands and task complexity varied between the studies; about half the tasks were assumed to be of low complexity, while six studies involved moderate to high task complexity. An example of an assumed low-complexity task involved lifting a box in a controlled setting from about 15 cm to knuckle height. In contrast, examples of high-complexity tasks included a series of various patient transfer tasks in a controlled setting and various care and health care tasks in real work environments. In terms of force demands, the majority of studies (79%) assumed to include or potentially include high force demands.

As shown in Table 8, the total number of participants was 444 (median = 20), of whom 43% were men, 51% were women, and 6% had unreported sex. In six studies, the greater majority (i.e., >60% of the participants) were men; in four studies, the greater majority were women; while in three studies, there was a balanced sex distribution (i.e., not more than 60% of any sex). Half of the studies (n = 7) included younger adults (mean age of 20–29 years); in four studies, the mean age was 30–39 years; and in two studies, the mean age was 40–49 years. Most of the studies (n = 9) included novice participants either unfamiliar with the tasks performed or students for whom the tasks were part of their training, while five studies included participants familiar with the task or similar tasks.

#### 3.2.2. Feedback Characteristics and Distribution, and Equipment

As indicated in Table 9, feedback effects were most commonly evaluated during the feedback delivery phase (10 studies) and immediately afterward (8 studies). Notably, only four studies [74,77,84,85] evaluated the retention effect beyond one week, while two studies [74,84] evaluated the retention effect beyond one month. The duration of the feedback training session varied considerably, ranging from one or two occasions of a few minutes to several weeks, as in the study by Ribeiro et al. [74], but typically ranged from about 10–15 min to an hour.

As shown in Table 10, the feedback trigger evaluated comprised of distinct cutoff thresholds at one or two levels (10 studies) or a constant audio tone that gradually increased in intensity with increased biomechanical load (4 studies). Notably, all the ambulatory systems used distinct thresholds. An alternative to the gradually increasing feedback intensity was to use two thresholds to further notify the user of increased biomechanical exposure, as was used in seven studies. Conversely, a single threshold level was used in only three studies.

In total, 307 participants received feedback training. The most common feedback modalities were auditory (9 studies), vibration (6 studies), and visual (1 study). In all studies, with the exception of Bootsman et al. [83], only one feedback modality was provided simultaneously. All studies evaluated feedback initiated automatically by the system rather than by the user. Additionally, corrective feedback was used in all studies, while Langenskiöld et al. [76] also used reinforcing feedback for half of the participants in combination with corrective feedback.

As shown in Table 11, motion capture data (derived from, e.g., IMUs and accelerometers) were used as input for the feedback in all studies, while muscle activity (derived from sEMG) was only used in the study by Agruss et al. [81]. The motion capture sensors are non-intrusive and positioned on the surface of the skin or clothing. Among the capture instruments, IMUs were the most common (9 studies, 64%), followed by optical (video-camera) motion capture systems (3 studies, 21%), while accelerometers and electromagnetic motion tracking devices were each used in one study. The use of a commercial system was stated as being used in one study (i.e., Ribeiro et al. [74]), while Agruss et al. [81] did not report the system used. In the remaining studies, custom solutions that applied a combination of commercial devices and custom-developed devices and programs were used. Fully ambulatory systems (i.e., with a greater possibility for field application) were evaluated in eight studies, meaning that they are not restricted to being used in one location at a time but can be worn by the user without setting up the equipment.

### 3.3. Effectiveness of Feedback in Real Work Environments

The findings of the studies assessing sensor-based augmented feedback in real work environments, categorized as high- and moderate-quality, are shown in Table 12. 

#### 3.3.1. Effect During Feedback Administration (Real Work Environments)

The effectiveness of sensor-based augmented feedback in reducing biomechanical exposure of the upper body in work-related manual handling tasks during its administration was evaluated in three studies, two of which were assessed as having high methodological quality. While Ribeiro et al. [74] observed modest and non-significant reductions in exposure for the intervention group, Lind et al. [77] reported considerable reductions, which were most pronounced during the second feedback training session and for peak exposures. For example, the proportion of time in trunk inclination ≥60° decreased by 89%, and trunk inclination ≥45° decreased by 80%, both of which were statistically significant and considered to have clinical relevance if maintained long-term. Similarly, the study by Bootsman et al. [83] indicated that the effect of feedback training increased from the first session to the second session, with a 25% significant reduction in the frequency of poor postures compared to the baseline.

Based on this, the current evidence for the effectiveness of sensor-based augmented feedback in reducing biomechanical exposure during administration is considered inconsistent in real work environments.

#### 3.3.2. Effect Directly After Feedback Administration (Real Work Environments)

The effectiveness of sensor-based augmented feedback directly after feedback administration was evaluated by Lind et al. [77] and Bootsman et al. [83]. While Lind et al. [77] reported considerable and statistically significant reductions in trunk inclination—such as a decreased proportion of time in trunk inclination ≥60° (67%) and ≥45° (61%)—Bootsman et al. [83] reported a tendency towards a decreased frequency of poor postures compared to the baseline, but this was not statistically significant.

Based on this, the current evidence for the effectiveness of sensor-based augmented feedback in reducing biomechanical exposure directly after administration is considered very limited in real work environments.

#### 3.3.3. Retained Effects: Very Short and Short Term (Real Work Environments)

The effectiveness of sensor-based augmented feedback after periods of up to one week and one month was evaluated in two high-quality studies by Ribeiro et al. [74] and Lind et al. [77]. Both studies observed no statistically significant effects of the feedback on episodes exceeding the lumbar postural threshold or trunk inclination. However, Lind et al. [77] observed a tendency for reduced peak angles (i.e., 95th and 99th percentiles) by up to 13% and a decreased portion of time spent above 60° trunk inclination (up to a 44% decrease), although these differences compared to baseline were not statistically significant.

Based on this, the assessment of the current evidence is that there is no evidence supporting the effectiveness of sensor-based augmented feedback after periods of up to one week and one month in real work environments.

#### 3.3.4. Retained Effects: Midterm and Long Term (Real Work Environments)

The effectiveness of sensor-based augmented feedback after periods of up to six months and more than six months was evaluated in a single high-quality study by Ribeiro et al. [74], for which no significant changes in frequency of exceeding the lumbar postural threshold due to the feedback were observed.

Based on this, the assessment of the current evidence is that there is no evidence supporting the effectiveness of sensor-based augmented feedback after periods of up to six months and more than six months in real work environments.

### 3.4. Effectiveness of Feedback in Controlled Settings

The findings of the studies assessing sensor-based augmented feedback in controlled settings categorized as high- and moderate-quality are shown in Table 13 and Table 14, respectively.

#### 3.4.1. Effect During Feedback Administration (Controlled Settings)

The effectiveness of sensor-based augmented feedback in reducing or maintaining a reduction in biomechanical exposure in manual handling in controlled settings during administration was evaluated in three high-quality studies [73,75,82,87] and three moderate-quality studies [70,76,86]. The four high-quality studies provided overall consistent, statistically significant reductions in posture exposure, as did the three moderate-quality studies. The effectiveness of sensor-based augmented feedback in reducing or maintaining a reduction in biomechanical exposure during manual handling in controlled settings was evaluated in four high-quality studies [73,75,82,87] and three moderate-quality studies [70,76,86]. The seven studies provided overall consistent, statistically significant reductions in posture exposure. For example, Boocock et al. [82] reported reductions in lumbosacral flexion and trunk flexion by 8% and 18.6%, respectively; Lind et al. [73] reported a 92% decrease in peak trunk inclination (i.e., 99th percentile) during the second feedback session; and Oppici et al. [87] reported a 12% reduction in peak sacro-lumbar flexion angle compared to the baseline.

Based on this, the assessment of the current evidence in controlled settings is that there is strong evidence supporting the effectiveness of sensor-based augmented feedback during administration.

#### 3.4.2. Effect Directly After Feedback Administration (Controlled Settings)

The effectiveness of sensor-based augmented feedback in reducing or maintaining a reduction in biomechanical exposure in manual handling in controlled settings directly after being administered was evaluated in three high-quality studies [73,84,87] and four moderate-quality studies [70,72,76,81]. The three high-quality studies provided consistent, statistically significant reductions in posture exposure, as did two of the moderate-quality studies. For example, Kamachi et al. [84] reported a 15% reduction in peak lumbar spine flexion, Lind et al. [73] reported a 34% decrease in peak trunk inclination (i.e., 99th percentile), and Oppici et al. [87] reported a 17% reduction in peak sacro-lumbar flexion angle compared to the baseline. The study by Agruss et al. [81] reported a statistically significant 25% reduction in lumbosacral compression force for the group receiving feedback based on acceleration and a 17% reduction tendency for the group receiving feedback based on EMG. Owlia et al. [72] reported a non-significant difference in median exposure (50th percentile) of lumbar spine flexion due to feedback training but a significant reduction in peak exposure by 29% for the 80th percentile and 36% for the 95th percentile.

Based on this, the assessment of the current evidence in controlled settings is that there is strong evidence supporting the effectiveness of sensor-based augmented feedback directly after it is administered when considering peak exposure.

#### 3.4.3. Retained Effects: Short and Midterm (Controlled Settings)

The effectiveness of sensor-based augmented feedback over periods of up to one and six months after administration was evaluated in one high-quality study by Kamachi et al. [84], and over periods of up to one month after administration in a moderate-quality study by Kernozek et al. [85]. The study by Kamachi et al. [84] presented a consistent, statistically significant reduction in posture exposure for the manual handling tasks included in the feedback training program. The study also assessed the effectiveness of feedback on a task not included in the training to evaluate the transfer of the training. For this task, a tendency toward reduced exposure was observed, but it was not statistically significant. In the study by Kernozek et al. [85], an average decrease in exposure of maximum spine moment (Nm) of 43% in forward flexion and 70% in lateral flexion was observed. However, the reporting of statistical tests involved the entire group of participants and not separately for the feedback group, making it difficult to conclude whether these large reductions were statistically significant.

Based on the results in the study by Kamachi et al. [84], the assessment of the current evidence in controlled settings is that there is limited evidence supporting the effectiveness of sensor-based augmented feedback of periods of up to one- and six months after administration for manual handling tasks included in the feedback training and no evidence for its effectiveness to transfer these effects to more complex tasks or tasks not included in the feedback training.

### 3.5. Summary of the Effectiveness of Feedback in Real Work Environments and in Controlled Settings

The summary of the consistency of the evidence for the effectiveness of sensor-based augmented feedback on manual handling in real work environments and controlled settings is shown in Table 15.

## 4. Discussion

### 4.1. General Summary of the Findings

This review adds new knowledge by synthesizing existing studies and evaluating the evidence on the effectiveness of sensor-based augmented feedback in reducing biomechanical exposure, such as posture, movements, and muscle activity, during manual handling tasks. Sixteen studies that evaluated the effectiveness of augmented feedback from sensor-based systems in reducing adverse biomechanical exposure of the upper body were identified and met the inclusion criteria. Fourteen of these studies were assessed as having high or moderate methodological quality and were included in the synthesis. Reductions in methodological quality scores were most commonly due to the lack of reporting participation rate of eligible persons, assessors not being blinded to participants’ group allocation, and insufficient justification of sample size.

Eleven studies were conducted in controlled settings and three in real work environments, with 13 employing (partly or fully) a cross-sectional design. A total of 444 participants were included, with a slight majority of women, although more studies had a majority of male participants. Half of the studies involved participants in their 20s, and nine studies included novice participants either unfamiliar with the tasks or students for whom the tasks were part of their training. Handling of box-type items was the most common task (8 studies). Most studies used audio feedback (9 studies) for providing feedback, followed by vibration feedback (6 studies) and visual feedback (1 study). All studies employed corrective feedback initiated by the system, with one study also using reinforcing feedback. The effect of feedback was most commonly evaluated during its delivery phase (10 studies) and immediately afterwards (8 studies). Feedback was rarely evaluated beyond one week (4 studies) or beyond one month (2 studies). IMUs were the most commonly used sensor to trigger feedback (9 studies), while three studies used video-based motion capture systems. The studies exhibited significant heterogeneity regarding the types of exposure and tasks evaluated, feedback administration programs and modalities (e.g., type of feedback and trigger), participant experience and age, and retention periods, limiting the feasibility of meta-analysis.

Considering the current evidence for the effectiveness of work technique training to reduce adverse biomechanical exposures in work-related manual handling utilizing sensor-based augmented feedback. When the feedback was evaluated on tasks performed in real work environments, the evidence for its effectiveness was considered to be inconsistent and very limited directly after administration. For longer periods after administration, ranging from one week to more than six months, there was no evidence demonstrating the effectiveness of the feedback. In controlled settings, there was strong evidence for its effectiveness during and immediately after administration, and limited evidence for effectiveness up to six months post-administration when considering the tasks included in the training.

### 4.2. General Interpretation of the Results

Compared to the recent reviews by Lind [67] and Lee et al. [69], which have partly overlapping scopes, this study includes five and twelve previously unreviewed studies for the assessment of methodological quality. Of these, four and eleven studies were incorporated into the synthesis of evidence for the effectiveness of augmented feedback. Additionally, five and nine studies assessed by Lind [67] and Lee et al. [69] for methodological quality did not meet the inclusion criteria of the current study due to differences in scope. The evidence in the current review for the effectiveness of sensor-based augmented feedback to reduce biomechanical load in work-related tasks ranges from no evidence to strong evidence, depending on the time frame when the feedback was last provided (i.e., during feedback administration—to more than six months after feedback administration) and the setting where the study was conducted (real work environment versus controlled setting). This finding contrasts with Lee et al. [69], who concluded that the evidence was limited for sensor-based augmented feedback in reducing biomechanical load related to the postures of the spine and arms. The results of the current review highlight the added value of synthesizing literature separately in terms of time frame and setting, allowing for a more granular assessment and facilitating the identification of research gaps.

Compared to Lind’s review [67], which exclusively targeted motion-based ambulatory systems but also included sedentary tasks such as computer work, the assessment of the strength of evidence mostly overlaps, except for two assessments. First, regarding the evidence for the effectiveness of feedback in real work environments for the time frame between one and six months (midterm), Lind [67] concluded that the evidence was inconsistent. In contrast, the current review found no evidence of its effectiveness. This difference can be attributed to the exclusion of a study by Bazazan et al. [56] that involved control room tasks. Second, the evidence for the effectiveness of feedback in controlled settings directly after its administration (within 8 h) was graded as moderate to high by Lind [67]. In contrast, the current review graded this evidence as strong, and the different grading was attributed to the studies by Oppici et al. [87] and Punt et al. [70].

#### Feedback Modalities and Sensors

The most common feedback modalities were auditory (9 studies) and vibration (6 studies), aligning with previous reviews by Lind [67] and Figueira et al. [50]. In contrast, Lee et al. [69] reported that visual and/or vibrotactile feedback was most frequently used.

IMUs were the most common source of input for augmented feedback, corresponding with the findings of Lind [67], but contrary to Lee et al. [69], where tri-axial accelerometers (without IMUs) were used in the majority of the studies. In the current review, tri-axial accelerometers (without IMUs) were only used in one study. The second most common sources were optical (video-camera) motion capture systems (3 studies, 21%), which contrasts with the review by Lind [67], who focused exclusively on ambulatory systems. Notably, only one of the included studies used sEMG as a source to trigger the feedback.

In most of the studies, the feedback was based on posture. Interestingly, velocity was less frequently used as input, especially in the ambulatory systems, despite the association between angular velocity of body segments and MSDs [89,90,91,92,93]. Recently, quantitative threshold levels based on the angular velocity of the wrist and arms have been proposed [94], offering potential as trigger points for augmented feedback in future systems. It should be noted that the threshold levels stressed are based on accelerometers, and the data need to be adjusted when using other types of sensors [95,96], for which there are some available conversion equations [97].

### 4.3. Limitations

#### 4.3.1. Limitations of the Evidence

Several aspects need to be considered when interpreting the results of this review. Firstly, the evidence of the effectiveness of sensor-based augmented feedback was based on studies evaluating its effectiveness in isolation from other training strategies, such as instruction from an instructor. In practical use, some may instead use sensor-based feedback combined with the guidance of an instructor.

The effectiveness of using an instructor has been reviewed by others [66], and in a few studies, it has been combined with sensor-based augmented feedback [98,99,100]. These latter studies were read in full but excluded from the current review, as it was judged that the instructor could have significantly impacted the results, and the effect of the sensor-based augmented feedback was not analyzed separately from the effect of the instructor. While the effectiveness of augmented feedback may increase when combined with instructor training, this is beyond the scope of the current review. The current literature on the effectiveness of sensor-based augmented feedback combined with instructor training in work-related tasks (such as manual handling) appears to be limited, as only three sources were identified, comprising a total of two studies [98,99,100], with only one study conducted in the last 20 years (i.e., Doss et al. [100]).

The interpretation of the strength of the evidence needs to consider the methodology used to grade it, which includes both the assessment of methodological quality and the criteria for grading the strength of the evidence. Notably, unlike some other approaches (e.g., [101]), studies with a non-RCT design could still fulfill the requirements for high methodological quality. Different grading systems, such as GARDE and others [102,103], may produce diverging results.

Compared to a recent rapid review on the effectiveness of augmented feedback in reducing adverse biomechanical exposure [67], the current review expanded its scope to include not only ambulatory systems but also non-ambulatory systems. This resulted in the inclusion of several additional studies that provided feedback from input via video-based motion capture systems. While the equipment used in these studies is mainly limited to research laboratory settings, the type of feedback evaluated can in principle be transferable to real work environments using more affordable video-based motion capture systems [104] or, e.g., IMUs [47].

A strength of the current review, compared to previous reviews, is the focus on one category of tasks, namely manual handling, rather than mixing heterogeneous tasks. Still, the manual handling tasks evaluated differ considerably between studies, such as in task complexity but potentially also in the inherent potential for improvement attribute due to constraints in the environment.

It should be emphasized that the evidence for the effectiveness of sensor-based augmented feedback refers specifically to its potential to reduce certain biomechanical exposures in a limited range of manual handling tasks. The results are constrained by their current applications, including specific training programs, types of feedback, and contexts. This effectiveness may not directly translate to most other manual handling tasks or contexts where the potential for reduced exposure through altered work techniques may be limited. Therefore, generalizing the results beyond these specific conditions is not supported by the current evidence, and the results should be interpreted with these limitations in mind.

The assessment of the evidence for the effectiveness of feedback is predominantly limited to the spine (excluding the cervical section). Therefore, the evidence of the effectiveness of feedback on individual body regions is outside the scope of this review.

The evaluation of the effectiveness of sensor-based augmented feedback training in this review was limited to short-term outcomes such as indirect (or partly direct) indicators of biomechanical load, including predicted lumbar spine compression force, lumbar spine moment, and postural angles (inclination, elevation, and flexion) of the spine or arms. While all of these factors have been associated with an increased risk of WMSDs [7,10,11,12,13,14,15,16,17,18,19,20,21,22,27,105,106,107,108,109], multiple task factors interact. Therefore, direct connections between the feedback trigger and risk of WMSDs should be interpreted with caution, and studies are needed to assess the effectiveness of sensor-based augmented feedback training on longer-term health outcomes such as WMSDs. As discussed by Lind [67], sensor-based feedback training often targets a reduction in exposure in one body region, which sometimes results in sub-optimal compensatory strategies where exposure is increased in other body regions, as observed by Lind et al. [86]. Examples of other negative side effects of augmented feedback include potential increased cognitive load [110].

#### 4.3.2. Limitations of the Review Processes

A rapid review methodology was followed; therefore, the process is less rigorous than that of systematic literature reviews. Rapid reviews have, however, a quicker review process, allowing the inclusion of more recent literature at the time of publication. The guideline for rapid reviews by Cochrane Rapid Reviews Methods Group [78] was followed, but with some deviations.

Two databases were used instead of the recommended three, and a single researcher performed the full screening of all titles and abstracts instead of two researchers screening 20%. Additionally, one reviewer, instead of two, screened the full-text articles for inclusion. The database search was restricted to articles published between 1 January 2020 and 9 June 2024. Articles published prior to 2020 were instead identified through 11 reviews/overviews, including five labeled as systematic literature reviews, five as reviews (scoping, narrative, or rapid), and one being an overview.

Given the large number of sources with overlapping scopes, it seems unlikely that a significant number of relevant studies were missed, although it cannot be entirely assured that one or a few relevant studies were not identified. Similar to a previous rapid review employing a similar mixed approach [67], the current rapid review identified several sources not included in several previous systematic reviews (e.g., [49,50,64,65,66,69]), highlighting the added value of combining multiple approaches in the literature search.

Furthermore, as per the Cochrane guidelines, the methodological quality assessment was performed by one reviewer, but this assessment was not verified by a second reviewer. The thresholds for methodological quality were the same as those used in the previously mentioned rapid review [67], which were more conservative than some previous reviews, where 33% of the methodological quality score was set as the threshold for moderate quality and 66% for high quality [111,112]. While there is no consensus on the most suitable thresholds, it was assumed that studies fulfilling only 1/3 of the methodological criteria could introduce too many biases, increasing the probability of both type-I and type-II errors. For this reason as well, studies assessed as having low methodological quality were excluded from the final analysis of the effectiveness of augmented feedback.

Additionally, contrary to some previous reviews (e.g., [69]), a restriction was set to exclude studies with fewer than eight participants per group unless they provided a power calculation justifying the sample size. This led to the exclusion of several studies (e.g., [113,114,115,116,117,118,119]). It cannot be ruled out that the exclusion of studies with a low sample size (not supported by power calculations, including studies having low methodological quality) could have influenced the grading of evidence by potentially introducing a type II error due to low statistical power. Conference papers (including conference abstracts) were also excluded (e.g., [113,114,120,121,122]) due to the often less rigorous peer-review process compared to scientific peer-reviewed journal articles.

### 4.4. Practical Implications and Future Research

This review identified several common methodological shortcomings that should be addressed to improve the quality of future evaluations on the effectiveness of sensor-based augmented feedback. The most frequently identified issues that reduced methodological quality scores were: lack of reporting participation rates of eligible persons, assessors not being blinded to participants’ group allocation, and insufficient justification of sample size. Other common issues included insufficient control of confounders and independent variables, particularly the work output per time unit, which was not always controlled or reported. While blinding assessors to participants’ group allocation might be less feasible, performing power calculations should be achievable, preferably based on pilot tests where the magnitude of changed exposure is estimated. Otherwise, the sources provided in the current review may be used as an alternative approach to make a rough estimation of the needed sample size. Notably, even larger sample sizes may be required when comparing the effects of two or more feedback characteristics, as evaluated by Langenskiöld et al. [76], Lim et al. [75], Punt et al. [70], and Agruss et al. [81].

Controlling work output per time unit is crucial, as it is otherwise difficult to evaluate potential differences in exposure attributed to the feedback. For example, in Ribeiro et al.’s RCT study [74], the work output per time unit was not reported, and large variations in exposure during the study period were reported for the control group as well.

If the variability in exposure differs considerably across workdays and sessions over the year, it might be difficult to assess potential changes in exposure attributed to the feedback if work output per time unit is not controlled. This challenge is especially significant when evaluating interventions in real work contexts of non-standardized tasks as opposed to standardized tasks performed in controlled environments. Therefore, future studies should consider various strategies to estimate work output, e.g., as conducted by Lind et al. [77] where the participants rated their work output compared to a normal workday, or (preferably) using reliable objective data at the individual level if available. Additionally, it is likely that the effectiveness of feedback varies across individual tasks. Hence, with a greater proportion of tasks—as is often the case in real work settings compared to controlled environments—the overall effectiveness might decline. It is also likely that other environmental factors may interfere with the feedback when applied in real work settings.

While this review focused on the effectiveness of sensor-derived augmented feedback in manual handling, multiple studies have focused on sedentary tasks, predominantly computer work [51,52,53,54,55,56,57,58,123,124] and odontology tasks [125,126,127,128]. Due to differences in activities compared to manual handling, the evidence for the effectiveness of augmented feedback from the current review cannot easily be transferred to such tasks. Therefore, future reviews are needed to synthesize its effectiveness in sedentary and predominantly static tasks such as computer work.

Future research should also explore how to optimize sensor-based augmented feedback, including which feedback types are most effective (e.g., modality: auditory, vibration, or visual; timing: concurrent, terminal, or fading). While some included studies targeted such comparisons, their number was too few and heterogeneous to draw conclusions or lacked statistical power (e.g., Langenskiöld et al. [76]).

Besides sensor feedback, tactile feedback from non-technical equipment such as athletic- or kinesio tapes [87,129,130,131] and dowels [132] has been used to reduce adverse spine posture in lifting. Studies indicate these methods have potential in reducing biomechanical exposure and retaining improvements, at least in the short term. For instance, the effectiveness of tactile feedback from sport leukotape was compared against concurrent sensor-based audio feedback, showing similar decreases in biomechanical exposures [87]. Therefore, future reviews should compare sensor-based feedback to other available sources of feedback.

The current review exclusively targeted sensor-based augmented feedback to improve biomechanical exposures in manual handling. Consequently, some otherwise relevant studies that combined augmented feedback from motion capture sensors or EMG were excluded, such as those by Lavender et al. [98,99] and Doss et al. [100], because they also used an instructor to guide the trainee and did not provide a separate analysis of the sensor-based feedback effect. Future reviews are needed to evaluate the potential added value of sensor-based feedback training assisted by an instructor in manual handling.

The potential effectiveness of sensor-based augmented feedback training is likely much lower compared to other measures aligned with the hierarchy of controls [47,133], such as engineering controls and organizational measures, which are generally more effective. Therefore, sensor-based augmented feedback training should not be used as the only measure to mitigate WMSDs but should (at best) be seen as a complementary strategy to other risk-reducing measures. Given indications of altered postural strategies and increased cognitive load due to augmented feedback, future efforts should focus on closely monitoring potential side effects, such as the transfer of load to other body parts and changes in cognitive exposure. Identifying task and work contexts where augmented feedback has the greatest potential to reduce adverse exposure and where it may be less effective or infeasible is also important. Additional barriers to implementation may include usability issues, such as ease of use and technology durability [30,134,135,136].

Only a limited number of studies have been performed in real work environments and evaluated the feedback with follow-up periods longer than 8 h. Therefore, there is a need to evaluate the effectiveness of sensor-based augmented feedback in real work environments over several days, weeks, and months. This information may contribute to much-needed knowledge about the optimum intervals for feedback training repetition. There is also a need for studies evaluating health effects, including MSDs and work-disabling conditions, in addition to short-term biomechanical exposures.

While there is strong evidence for the effectiveness of sensor-based augmented feedback in reducing exposure in manual handling during and directly after administration in controlled settings, the long-term effects as well as effectiveness in real work environments remain largely unexplored. This limits the recommendation for sensor-based augmented feedback as a risk-reducing measure in work-related manual handling. Consequently, more research is warranted to assess its potential in reducing biomechanical exposure and work-related musculoskeletal disorders (WMSDs) in manual handling.

## 5. Conclusions

In this rapid review, the current evidence for the effectiveness of work technique training to reduce adverse biomechanical exposures in work-related manual handling utilizing sensor-based augmented feedback was assessed. Sixteen studies meeting the inclusion criteria for assessment of methodological quality were identified, of which seven were assessed as having high methodological quality and seven as having moderate methodological quality, and these were included in the synthesis of the evidence. The most frequent reasons for reductions in methodological quality scores were the lack of reporting participation rate of eligible persons, assessors not being blinded to participants’ group allocation, and insufficient justification of sample size. Of the 14 included studies, three were conducted in real work environments and eleven in controlled settings. Most studies used auditory feedback (n = 9), followed by vibration feedback (n = 6). All studies evaluated corrective feedback initiated by the system, whereas reinforcing feedback was additionally evaluated in one study.

In real work environments, the current evidence for the effectiveness of sensor-based augmented feedback in reducing biomechanical exposure during administration is considered inconsistent and very limited directly after administration. For longer periods after administration, ranging from one week to more than six months, there is currently no evidence demonstrating the effectiveness of the feedback.

In controlled settings, there is strong evidence for its effectiveness during and immediately after administration, and limited evidence for effectiveness up to six months post-administration when considering the tasks included in the training. When considering the ability to transfer the reduced exposures following feedback training to other tasks, there is no evidence demonstrating its effectiveness.

The existing literature on the retained effects of work technique training using sensor-based augmented feedback to reduce adverse biomechanical exposures in work-related manual handling over periods extending beyond a workday is limited. This scarcity restricts recommendations for or against its use as a measure to mitigate adverse biomechanical exposures in work-related manual handling. Consequently, the publication of new research could potentially alter the grading of evidence for its effectiveness, particularly studies conducted in real work contexts of short- and long-term, but also short- and long-term studies in controlled settings. To improve the methodological quality of future studies, it is essential to address the identified gaps in reporting or performing power calculations and reporting of participation rates. Additionally, when feasible, blinding of assessors to participants’ group allocation should be implemented. Given the limited comparison of different feedback modalities and feedback training programs, research evaluating the effectiveness of various feedback modalities and training programs is also identified as an important research gap to address.

## Figures and Tables

**Table 1:** The eligibility criteria for the inclusion and exclusion of studies.

Eligibility Criteria	Descriptions
Studies evaluating sensor-based instruments or systems that monitor biomechanical exposure targeting the upper body and provide augmented feedback to the wearer based on this information.	Various biomechanical exposures are targeted, including posture, movement, muscle activity, etc. The primary body segments targeted include the neck (e.g., head inclination), spine (cervical to lumbosacral segments), arms (upper and lower), and wrists/hands. Only instruments or systems providing direct feedback to the wearer were considered. For example, evaluations on the effectiveness of an instructor assisted by sensor-based data are outside the scope. The focus on sensor-based augmented feedback excludes other types of augmented feedback, such as from athletic tapes.
Studies aim to reduce adverse biomechanical exposure with the long-term objective of preventing or reducing work-related musculoskeletal disorders (WMSDs).	This study should report (at least briefly) how the reduction of the targeted biomechanical exposure can potentially mitigate adverse health effects, including WMSDs. Studies aiming to increase biomechanical exposure to achieve health benefits, such as interrupting prolonged sustained postures with increased physical activity, are excluded.
Studies evaluating augmented feedback on manual handling tasks performed in real work environments or those that simulate work-related manual handling tasks or single operations in controlled settings.	Manual handling operations encompass tasks such as lifting, pushing, pulling, and holding. This review excludes studies focused on predominantly sedentary jobs or tasks involving a significant proportion of static (isometric) postures, such as computer typing, dentistry, or surgical work. Additionally, the emphasis on work-related manual handling tasks excludes studies involving the manipulation of non-physical (virtual) objects.
Studies evaluating augmented feedback on adults (18–67 years) from regular working populations or student populations.	If the sample includes participants older or younger than 18–67 years, the data on the effect of feedback must be reported separately for those aged 18–67 years. The focus on regular working populations and student populations means that specific patient populations, such as those with medical conditions that hinder task performance, are excluded.
Studies evaluating augmented feedback on at least 8 participants per group receiving the feedback and where the effect of the feedback is tested statistically	A sample size of fewer than 8 participants per group is acceptable only if justified by power calculations, including descriptions of the assumed effect size. The number of participants refers to those included in the final analysis.

**Table 2:** Criteria for assessing the strength of evidence (based on Lee et al. [66] and Lind [67]).

Strength of Evidence	Criteria
Strong evidence	Consistent findings among three or more studies of at least moderate quality, including at least two of high quality.
Moderate evidence	Consistent findings among two or more studies of at least moderate quality, including at least one of high quality.
Limited evidence	Findings from at least one study of high quality or two studies of moderate quality.
Very limited evidence	Findings from one study of moderate quality.
Inconsistent evidence	Inconsistent findings among multiple studies, such as one or multiple studies of at least moderate quality reporting significant results, whereas one or multiple studies of at least moderate quality reporting no significant results.
Conflicting evidence	Conflicting results between studies, such as one or multiple studies of at least moderate quality reporting significant results in one direction, whereas one or multiple studies of at least moderate quality reporting significant results in the other direction.
No evidence	Insignificant results derived from multiple studies of high or moderate quality.

**Table 3:** Feedback retention categories, modified based on the duration classification by Lind [67].

Duration Classification	Criteria (Time Elapsed After Feedback Administration)	Abbreviated Names
During feedback	Simultaneous to feedback administration	During feedback
Directly after	Directly after, and up to 8 h after	Directly after (≤8 h)
Very short term	>8 h, and up to ≤1 week after	Very short term (≤1 week)
Short term	>1 week, and up to ≤1 month after	Short term (≤1 month)
Midterm	>1 month, and up to <6 months after	Midterm (<6 months)
Long term	6 months or more after	Long term (≥6 months)

**Table 4:** Methodological quality assessment using the NHLBI tool for observational cohort or cross-sectional studies (see Table A3 for a full description of the criteria). High-quality studies are indicated by green, moderate-quality studies by yellow, and low-quality studies by red.

Study	Criteria														Quality
	1	2	3	4	5	6	7	8	9	10	11	12	13	14	
Agruss et al. [81]	1	1	NR	1	NR	1	1	1	1	0	1	NR	1	1	Moderate
Boocock et al. [82]	1	1	NR	1	1	NA	1	1	1	1	1	NR	1	1	High
Bootsman et al. [83]	1	1	NR	NA	NR	1	1	1	0	1	1	NR	1	0	Moderate
Brandl et al. [71]	1	1	NR	1	NR	0	0	1	1	0	0	NR	1	0	Low
Kamachi et al. [84]	1	1	NR	1	1	1	1	1	1	1	1	NR	1	1	High
Kernozek et al. [85]	0	0	NR	1	NR	1	1	1	1	0	1	NR	1	1	Moderate
Langenskiöld et al. [76]	1	1	NR	NA	NR	1	1	1	0	1	1	NR	1	0	Moderate
Lim et al. [75]	1	1	NR	NA	NR	1	1	1	1	1	1	NR	1	1	High
Lind et al. [86] Sens	1	1	NR	NA	NR	1	1	1	0	1	1	0	1	0	Moderate
Lind et al. [73] AE	1	1	NR	NA	NR	1	1	1	1	1	1	0	1	1	High
Lind et al. [77] Erg	1	1	1	NA	NR	1	1	1	1	1	1	0	0	1	High
Oppici et al. [87]	1	1	NR	NA	NR	1	1	1	1	1	1	NR	1	1	High
Owlia et al. [72]	1	1	NR	1	NR	1	1	1	0	1	1	NR	1	0	Moderate
Punt et al. [70]	1	0	NR	NR	NR	1	1	1	1	1	1	NR	1	1	Moderate
Ribeiro et al. [88]	1	1	NR	0	NR	1	1	1	0	1	0	0	0	0	Low
Ribeiro et al. [74]	1	1	NR	1	1	1	1	1	1	1	1	1	1	1	High

Notes: 1: criteria fulfilled; 0: criteria not fulfilled; NA: not applicable; NR: not reported. Questions: 1. Clear research question; 2. Clear study population; 3. Participation rate; 4. Subjects recruitment; 5. Justified sample size; 6. Temporality of exposure(s) and outcome(s); 7. Sufficient time 8. Dependent variable scaling; 9. Independent variables treatment; 10. Assessment of dependent variable; 11. Assessment of dependent variable; 12. Blinding; 13. Loss to follow-up; 14. Control of confounders.

**Table 5:** Methodological quality assessment using the NHLBI tool for controlled intervention studies (see Table A4 for a full description of the criteria). High-quality studies are indicated by green and low-quality studies by red.

Study	Criteria														Quality
	1	2	3	4	5	6	7	8	9	10	11	12	13	14	
Ribeiro et al. [88]	1	0	0	0	NR	0	0	1	NR	NR	0	0	NR	1	Low
Ribeiro et al. [74]	1	1	1	1	1	1	1	1	NR	NR	1	1	1	1	High

Notes: 1: criteria fulfilled; 0: criteria not fulfilled; NR: not reported. Questions: 1. Study description; 2. Randomization; 3. Concealed allocation; 4. Blinding (providers and participants); 5. Blinding (assessors); 6. Baseline characteristics; 7. Endpoint dropout; 8. Endpoint dropout (between groups); 9. Adherence; 10. Confounding interventions; 11. Quality of outcomes assessment; 12. Justified sample size; 13. Prespecified analysis; 14. Group assignment.

**Table 6:** Study characteristics and targeted outcomes.

Study	Targeted Outcome	Study Design	Control Group	Setting
Agruss et al. [81]	Reduce peak lumbosacral compression	CS	Yes	Cont.
Boocock et al. [82]	Reduce lumbosacral posture and trunk flexion	CS	Yes	Cont.
Bootsman et al. [83]	Reduce episodes of lower back flexion	CS	No	Real
Kamachi et al. [84]	Reduce time in end-range lumbar spine flexion	CS/SLN	Yes	Cont. ^1^
Kernozek et al. [85]	Reduce peak lumbosacral moment	CS	Yes	Cont.
Langenskiöld et al. [76]	Reduce time in adverse trunk inclination and arm elevation	CS	No	Cont.
Lim et al. [75]	Reduce sagittal trunk flexion angles	CS	No	Cont.
Lind et al. [86]	Reduce time in adverse arm elevation	CS	No	Cont.
Lind et al. [73]	Reduce time in adverse trunk inclination and arm elevation	CS	No	Cont. ^2^
Lind et al. [77]	Reduce time in adverse trunk inclination	CS/SLN	No	Real
Oppici et al. [87]	Reduce spine flexion	CS	No	Cont.
Owlia et al. [72]	Reduce peak lumbar spine flexion	CS	Yes	Cont. ^1^
Punt et al. [70]	Reduce low-back load (L5/S1 net moment, trunk inclination, and lumbar flexion)	CS	Yes	Cont.
Ribeiro et al. [74]	Reduce occurrence of trunk inclination	cluster RCT	Yes	Real

Notes: CS: cross-sectional; SLN: semi-longitudinal; RCT: randomized control trial; Real: real work environment; Cont: controlled setting; ^1^ HomeLab at Toronto Rehabilitation Institute; ^2^ training facilities in a real work setting.

**Table 7:** Description of the manual handling tasks in the studies, force demands, and task complexity.

Study	Force Demands	Task Complexity	Tasks	Description
Agruss et al. [81]	H	L	Lifting	Symmetrical sagittal plane lifts of boxes (“maximum safe load” ^1^) from pallet height to knuckle height at 30-s intervals.
Boocock et al. [82]	H	L	Lifting and lowering	Lifting and lowering a 13-kg box (30 × 25 × 25.5 cm) with handles (10 lifts/min) from a platform (height: 15 cm) to an upright standing position, holding it still, and then lowering the box back to the platform.
Bootsman et al. [83]	L–H	H	Health care and home care tasks	Regular intensive care and home care tasks.
Kamachi et al. [84]	H	M–H	Patient transfer	A series of simulated patient transfer activities of a patient actor (87 kg), e.g., transfer patient from bed to wheelchair, from wheelchair to couch, and assist patient to stand, don pants, and transfer to wheelchair. Additional skill transfer task: patient sling insertion task.
Kernozek et al. [85]	H	L	Lifting and lowering	Lifting cases of bananas (mean: 18 kg) and various items (13.6–18.1 kg). Items were lifted from the pallet on the floor to a 2.1-m height location and from the 2.1-m height location to the pallet.
Langenskiöld et al. [76]	L–M	L–M	Office-type of manual handling	Organizing 38 documents, lifting 8 large empty boxes, relocating 8 ring binder, and lifting 10 small empty boxes.
Lim et al. [75]	M–H	M	Construction activities	Lifting pouches (25 × 36 cm, 4.5 kg) from floor height, carrying them 1.2 m, and lowering them. Transferring the pouches using a shovel to a location 1.2 m away. Simulated rebar tying tasks.
Lind et al. [86]	L	L	Mail (letter) sorting	Sorting 30 randomly ordered letters (marked 0–9) to their corresponding letter tray (marked 0–9).
Lind et al. [73]	L–M	M–H	Order picking	Simulated order-picking task resembling real order-picking, with items (0.1–3.1 kg) located in seven positions (heights: 0.15–1.3 m).
Lind et al. [77]	M–H	H	Order picking	Sorting of packages (3–15 kg, 3–6 packages/min) from a container to a storage container (height: 0.3–1.1 m). Handling of containers was also included (including pushing) and registering each handled item.
Oppici et al. [87]	H	L	Lifting and lowering	Lifting (symmetrical sagittal plane lift) a 7.5-kg box from floor height to knuckle height and lowering it back to floor height (10 lifts/min).
Owlia et al. [72]	H	M–H	Patient transfer	A series of simulated patient transfer activities of a patient actor (82 kg), e.g., transfer patient from bed to wheelchair, from wheelchair to couch, and assist patient to stand, don pants, and transfer to a wheelchair.
Punt et al. [70]	H	L	Lifting and lowering	Lifting and lowering a 10-kg box to three locations: a left/center/right position and two depth (far and nearby) positions.
Ribeiro et al. [74]	L–H	H	Health care activities	Regular healthcare activities are performed within aged care institutions and hospitals.

Notes: assumed force demands and task complexity, low (L), moderate (M), high (H); ^1^ maximum safe load refers to a load weight of less than 24 kg and results in an individually predicted compression force of less than 3.4 kN.

**Table 8:** Participants’ characteristics and eligibility criteria.

Study	Participants: Sex, Age (Mean, SD)	Eligibility (Health Status)
Agruss et al. [81]	28 college students (10 men; 18 women), 23.2 (3.4) years. Verbal acceleration feedback group: n = 9; 24.1 (4.8) years. EMG feedback group: n = 10; 22.8 (2.5) years. Control group: n = 9; 22.7 (2.4) years.	Not having acute or chronic back pain.
Boocock et al. [82]	36 university students ^1^ (sex: NR). Feedback group: (n = 18); 25.7 (4.6) years. Control group: (n = 16); 25.6 (5.1) years.	No back injury or complaint in the past 6 months; no history of spinal surgery; without any cardiovascular or neurological conditions; no existing musculoskeletal injury. Other ^2^: not experienced in manual handling or performed regular handling in their work.
Bootsman et al. [83]	13 nurses (all women); 40 (14) years.	No LBP. Other: not having a sedentary job
Kamachi et al. [84]	20 participants (10 men; 10 women). Feedback group (5 female; 5 male); 24 (4) years. Control group (5 female; 5 male); 24 (3) years.	No back pain in the last 6 months or any MSDs or issues related to the spine. Other: no previous experience in caregiving or healthcare; able to understand and speak English.
Kernozek et al. [85]	22 warehouse workers (all men); age: NR. Feedback training group (n = 11); Control group (n = 11).	Health status: NR. Other ^2^: regularly performing warehouse lifting or lowering tasks.
Langenskiöld et al. [76]	10 participants ^3,4^ (2 men; 8 women); 43.9 (12.0) years.	Not having pain or restrictions in movement.
Lim et al. [75]	14 participants (all men); 26 (5) years.	Without MSDs. Other: 18–35 years age; no previous experience of construction; no previous training on safe construction work techniques.
Lind et al. [86]	16 university staff/students (7 men; 9 women); 25 (8) years.	No musculoskeletal discomfort or disorders that could hinder the manual handling task.
Lind et al. [73]	15 ^5^ warehouse workers (12 men; 3 women); 39 (12) years.	No musculoskeletal discomfort or disorders that could hinder the manual handling task.
Lind et al. [77]	15 warehouse workers (14 men; 1 women) ^6^; 31 (12) years.	No disorders or pain that prevent performing daily work. Other: currently working as order picker.
Oppici et al. [87]	20 university students (13 men; 7 women); 30 ± 6 years.	Not having back injury or pain in the last year, undergoing spinal surgery, or any cardiovascular, neurological or musculoskeletal condition at the time of the study, or allergy to adhesives.
Owlia et al. [72]	20 participants (10 men; 10 women). Feedback training group: (6 men; 4 women); 28 (6) years. Control group: (4 men; 6 women); 25 (3) years.	^2^ No history of back pain in the last six months and no musculoskeletal issues related to the spine. Other ^2^: adults (i.e., ≥18 years); having no formal training in caregiving or patient handling; able to understand and speak English.
Punt et al. [70]	Control group: 14 participants (7 men and 7 women); 23.7 ± 8 years. Feedback moment group: 29 participants (22 men and 7 women); 25.7 ± 4 years. Feedback inclination group: 28 participants (13 men and 15 women); 24.9 ± 7 years. Feedback lumbar flexion group: 21 participants (15 ^7^ men and 7 ^7^ women); 25.9 ± 10 years.	^2^ No recent history of low back pain. Other ^2^: no previous knowledge about the biomechanics of lifting; not having participated in any other studies related to lifting and biomechanics.
Ribeiro et al. [74]	130 healthcare workers (20 men; 110 women); 45 (13) years. Feedback group (10 men; 53 women); 48 (range: 37–55) years. Control group (10 men; 57 women); 47 (range: 32–56) years.	Performing regular work activities without any limitations such as due to LBP or LBP symptoms. Other: adult health care worker; working at least 20 h/week.

Notes: ^1^ 31 participants’ data were analyzed, i.e., 15 of 18 participants in the feedback group completed the full session and all participants in the control group. ^2^ not explicitly reported as inclusion criteria; ^3^ data analyzed for 9 participants; ^4^ nine administrative office workers and one industrial manual handler; ^5^ the data of 2 participants were excluded from the final analysis due to technical issues; ^6^ data collection were performed on 20 participants but only 15 completed all sessions and were included in the final analysis; ^7^ the numbers reported by Punt et al. [70] do not add up.

**Table 9:** Feedback evaluation and distribution.

Study	Feedback Evaluation	Feedback Distribution
Agruss et al. [81]	During feedback ^1^ Very short term (≤1 week)	Baseline—All lifting two 5-min sets (each 10 lifts) without feedback; instructions from video on the basics of safe lifting mechanics (all participants) Week 1—CG: 40 lifts without feedback; FBGs A and B: 10 lifts without feedback; 10 lifts (100% feedback); 10 lifts (50% feedback); 10 lifts without feedback. Week 2—CG: 40 lifts without feedback; FBGs A and B: 10 lifts without feedback; 5 lifts (100% feedback); 15 lifts (33% feedback); 10 lifts without feedback. Week 3—All: lifting two 5 min sets (each 10 lifts) without feedback Feedback session duration: ~15 min
Boocock et al. [82]	During feedback	Order (no baseline): Lifting for 20 min (FBG: with feedback; CG: without feedback) Feedback session duration: ~20 min
Bootsman et al. [83]	During feedback Directly after (≤8 h)	Order: Baseline (30 min, no feedback); Feedback session 1 (60 min, feedback); Retention test session (60 min, no feedback); Feedback 2 session (60 min, feedback ^3^) Feedback session duration: ~120 min
Kamachi et al. [84]	Directly after (≤8 h) ^4^ Short term (≤1 month) Midterm (≤6 months)	Session 1: FBG + CG (no feedback); FBG + CG video training Sessions 2 and 3: FBG (100% feedback); CG: (no feedback) Sessions 4 and 6: FBG + CG (no feedback) Sessions 6 and 7: FBG (50% feedback); CG (no feedback) Session 8: FBG + CG (no feedback) Retention tests (sessions 9 and 10): previous tasks and a new task to test the skill transfer Sessions 9 (after 2 weeks): FBG + CG (no feedback) Sessions 10 (after 2 months): FBG + CG (no feedback) Feedback session duration: ~60 min. Session duration: about 15 min each for sessions 1–8 (sessions 1–4 performed on day 1 and sessions 5–8 on day 2)
Kernozek et al. [85]	Short term (≤1 month)	Week 1 (baseline): Lifting ^2^ (both groups: without feedback) Weeks 2 and 4: Lifting ^2^ (feedback group: with feedback; control group: not lifting) Weeks 6: Lifting ^2^ (both groups: without feedback) Feedback session duration: ~30 min
Langenskiöld et al. [76]	During feedback Directly after (≤8 h)	Order: Practice session; baseline (4–6 min, no feedback); feedback session (8–12 min, feedback); retention session (4–6 min, no feedback). Feedback session duration: ~8–12 min
Lim et al. [75]	During feedback	Random order: 3 feedback sessions each performed in 3 tasks. Feedback condition: no feedback; feedback from device on the back of the wrist. Tasks: lifting/lowering (mean: 3.4 min); shoveling (mean: 7.2 min); rebar tying (mean: 6.9 min) Feedback session duration: ~35 min
Lind et al. [86]	During feedback	Order: Practice session (no feedback); (b) baseline (no feedback); ergonomics instruction session 1 (no feedback); feedback session 1 (feedback); ergonomics instruction session 2 (no feedback); feedback session 2 (feedback). Session duration: all sessions but practice session (~1 min) Feedback session duration: ~2 min
Lind et al. [73]	During feedback Directly after (≤8 h)	Order: Practice session (no feedback); Baseline (no feedback); Feedback session 1 (feedback); Feedback session 2 (feedback); post-Feedback session (no feedback). Session duration: all sessions but practice session (~6 min) Feedback session duration: ~12 min
Lind et al. [77]	During feedback Directly after (≤8 h) Very short term (≤1 week) Short term (≤1 month)	Order: Baseline (no feedback): Feedback session 1 (2 days after baseline; feedback); Feedback session 2 (~7 days after baseline; feedback); post-Feedback session (directly after Feedback session 2; no feedback); Retention session 1 (~1 week after Feedback session 2; no feedback); Retention session 2 (~3 weeks after Feedback session 2; no feedback). Session duration: Baseline (~45 min); Feedback sessions and post-Feedback session (~30 min); Retention sessions (~45 min) Feedback session duration: ~60 min
Oppici et al. [87]	During feedback Directly after (≤8 h)	All conditions: 30 lifts/lower; 6 min rest; retention test (5 lifts/lower) Order (2 min break between condition): Baseline, Feedback condition (A or B), Feedback condition (B or A) Feedback was not provided during retention test Feedback session duration: ~3 min each with audio- and tactile feedback
Owlia et al. [72]	Directly after (≤8 h) ^3^	Day 1—Session 1 (no feedback); Video training (only FBG); Session 2 (no feedback); Sessions 3 and 4 (FBG: feedback; CG no feedback); Day 2—Session 5 (no feedback); Sessions 6 and 7 (FBG: feedback; CG no feedback); Session 8 (no feedback) Session duration: session 1–7 (~10 min) Feedback session duration: ~about 40 min
Punt et al. [70]	During feedback Directly after (≤8 h)	All sessions: 12 lifts/lower Session 1 (Baseline): no feedback Sessions 2 and 3: feedback for feedback groups; no feedback for the control group. Session 4 (retention test): no feedback Feedback session duration: NR
Ribeiro et al. [74]	During feedback Very short term (≤1 week) Short term (≤1 month) Midterm (≤6 months) Long term (≥12 months)	Baseline Intervention (for 4 weeks) Retention tests after 1 week, 1 month, 3 months, 6 months, 12 months. Feedback session duration: 4 work weeks

Notes: Abbreviations: CG, control group; FBG. Feedback group; NR, not reported; ^1^ not analyzed statistically; ^2^ series of six lifts in different conditions; ^3^ visual feedback and note-taking were used in combination with auditory and vibration feedback; ^4^ the effect observed while providing feedback was reported but not evaluated statistically and was therefore excluded from this analysis.

**Table 10:** Feedback characteristics, targeted body region(s), and feedback trigger.

Study	Feedback Type	Feedback Modality	Primary Body Region(s)	Feedback Trigger
Agruss et al. [81]	System-initiated Corrective fading Concurrent or terminal ^1^	A: audio B: audio (verbal)	Lumbosacral spine	Gradually increased feedback intensity: Group A: muscle activity (electromyographic) Group B: acceleration index (difference between the dynamically and statically determined lumbosacral peak compression forces)
Boocock et al. [82]	System-initiated Concurrent corrective	Audio	Lumbosacral spine	1 feedback level: >80% of maximum lumbosacral range-of-motion
Bootsman et al. [83]	System-initiated Concurrent cumulative) corrective	Audio and Vibration ^2^ + Visual ^3^	Lumbosacral spine	1 feedback level: >20° lower back flexion for >1.5 s (max 1 notification/5 min)
Kamachi et al. [84]	System-initiated Concurrent and fading ^4^ corrective	Audio	Lumbosacral spine	2 feedback levels: 20° less than 70% max forward lumbar flexion (intermittent tone); >70% of max forward lumbar flexion (continuous tone).
Kernozek et al. [85]	System-initiated Concurrent corrective	Audio	Lumbosacral spine	Gradually increased feedback intensity with increased spinal moments (L5/S1)
Langenskiöld et al. [76]	System-initiated Terminal corrective and reinforcing	Vibration	Spine (thoracic–lumbosacral) Upper arm	1 feedback level per body region: >30° trunk inclination for >10% of the time. >30° arm elevation for >30% of the time.
Lim et al. [75]	System-initiated Concurrent (cumulative) corrective	Vibration	Spine (thoracic–lumbosacral)	2 feedback levels: >45° trunk inclination (3 intermittent vibrations). 3 s continuous vibration if the criteria was reached >2 times within 2 min.
Lind et al. [86]	System-initiated Concurrent corrective	Vibration	Upper arm	2 feedback levels: Arm elevation ≥ 30° and ≥60°
Lind et al. [73]	System-initiated Concurrent corrective	Vibration	Spine (thoracic–lumbosacral) Upper arm	2 feedback levels per body region: Arm elevation ≥ 30° and ≥60° Trunk inclination ≥ 20° and ≥45°
Lind et al. [77]	System-initiated Concurrent corrective	Vibration	Spine (thoracic–lumbosacral)	2 feedback levels: Trunk inclination > 30° and >45°
Oppici et al. [87]	System-initiated Concurrent corrective ^5^	Audio	Lumbosacral spine	Gradually increased feedback amplitude and modulation frequency with increased spine flexion angle.
Owlia et al. [72]	System-initiated Concurrent corrective	Audio	Lumbosacral spine	2 feedback levels: 20° less than 70% of maximum forward lumbar flexion; 70% of maximum forward lumbar flexion.
Punt et al. [70]	System-initiated Concurrent corrective	Audio	Lumbosacral spine	Gradually increased feedback intensity. A ^6^ 80% of the average peak sagittal plane moments during baseline. B ^7^ 80% of the average peak trunk inclination angles during baseline. C ^8^ 70% of the average of the observed peak lumbar flexion angles during baseline
Ribeiro et al. [74]	System-initiated Concurrent cumulative corrective	Audio	Lumbosacral spine	2 feedback levels: ≥45° lumbopelvic forward bend (continuous > 5 s) ≥45° lumbopelvic forward bending (occurring within 25 s after condition 1).

Notes: ^1^ group A: concurrent feedback and group B: terminal feedback; ^2^ condition 1 (audio and vibration feedback); ^3^ condition 2 (audio, vibration, and visual feedback); ^4^ 2 variations: feedback given each time the criteria were met (i.e., 100%), and fading where provided half the time the criteria were met (i.e., 50%); ^5^ group B received tactile feedback from Sport leukocyte, but this is not included in this synthesis. ^6^ Moment feedback group; ^7^ Inclination feedback group; ^8^ Lumbar flexion feedback group.

**Table 11:** Equipment for collecting and analyzing exposure data.

Study	Equipment (Exposure Analysis; Feedback Trigger)	Motion Sensor	Ambulatory
Agruss et al. [81]	Group A: NR Group B: Custom	Prototype version of the video analysis system, the V-Task. 6 reflective markers on the wrist, elbow, shoulder, hip, knee, and ankle joint. Group A: additionally, an EMG-system (name and location: NR)	No
Boocock et al. [82]	Custom: custom-designed software (LabView)	2 IMUs (Shimmer Sensing, Dublin, Ireland) Location: 1st lumbar spinous process and sacral body (S1)	Partly
Bootsman et al. [83]	Custom: smartphone Android application	2 IMUs (LSM9DSO, STMicroelectronics, Stockholm, Sweden) Location: 1st and 5th lumbar spine vertebrae	Yes
Kamachi et al. [84]	Custom: PostureCoach v0.2	2 IMUs (MTi-3, Xsens Technologies, Enschede, The Netherlands) Location: (back) thoracic vertebrae (T10) and approx. to sacrum	Yes
Kernozek et al. [85]	Custom and commercial: motion monitor software (Innovative Sports Training, Inc., Chicago, IL, USA); Custom Matlab programs (Version 6.5, The Mathworks Inc., Natick, MA, USA); Motion Monitor software. Auditory feedback was controlled by the LiftTrainerTM software.	Ascension Electromagnetic Tracking Device (Ascension Technology Corporation, Burlington, VT, USA) Sensor location: forearms, upper arm, back of the head, cervical (C4) and sacral (S1) regions of the spine.	No
Langenskiöld et al. [76]	Custom: Smartphone Android application (ErgoRiskLogger)	2 IMUs (LPMS-B2 IMU, LP Research, Tokyo, Japan) Location about at the level of 1–2 thoracic vertebrae and distal part of m. deltoideus.	Yes
Lim et al. [75]	Custom: custom-designed software with hardware Raspberry Pi 3 board and PC	4 IMUs (Mbientlab MetaMotionR+) Location: 6th thoracic vertebra, right thigh, right shin, and dominant wrist	Partly
Lind et al. [86]	Custom: smartphone Android application (ErgoRiskLogger)	1 IMU (LPMS-B2 IMU, LP Research, Tokyo, Japan) Location: distal part of m. deltoideus	Yes
Lind et al. [73]	Custom: smartphone Android application (ErgoRiskLogger)	2 IMUs (LPMS-B2 IMU, LP Research, Tokyo, Japan) Location: 1–2 thoracic vertebrae, and distal part of m. deltoideus	Yes
Lind et al. [77]	Custom: smartphone Android application (ErgoRiskLogger)	1 IMU (LPMS-B2 IMU, LP Research, Tokyo, Japan) Location: 1–2 thoracic vertebrae	Yes
Oppici et al. [87]	Custom: Visual3D software (CMotion, Inc.) and custom script in MATLAB (The Mathworks Inc., Natick, MA, USA); Pure Data via Open Sound Control protocol.	10-camera motion capture system (Qualisys AB, Gothenburg, Sweden) with 36 reflective markers attached to the trunk, pelvis, thighs, shanks, and feet.	No
Owlia et al. [72]	Custom: PostureCoach v0.2	2 IMUs (MTi-3, Xsens Technologies, Enschede, The Netherlands) Location: 10th thoracic vertebrae and approx. to sacrum	Yes
Punt et al. [70]	Custom: custom-made Matlab (The Mathworks Inc., Natick, MA, USA) program	3 camera arrays of a 3D motion capture system (Optotrak Certus system; Norton Digital Inc., Waterloo, ON, Canada). Light-emitting diodes markers attached: both shanks and thighs, pelvis (sacrum), and thorax (T6 spinous process).	No
Ribeiro et al. [74]	Commercial: Spineangel (Movement Metrics Ltd., Hamilton, New Zealand)	1 triaxial accelerometer (Spineangel) Location: lateral around the hip	Yes

Notes: NR: not reported; IMU: inertial measurement unit.

**Table 12:** Summary of results from high- and moderate-quality studies in real work environments: Statistically significant decreases in exposures are indicated by green, non-statistically significant decreases by yellow, and non-statistically significant increases by orange, with *p*-values shown in parentheses.

Study	Reported Effects					
	**High quality studies**					
Lind et al. [77]	Median intra-individual differences in trunk inclination (feedback condition vs. baseline)					
	Distribution (angle, °)			Proportion of the time		
	During feedback					
		1st occasion	2nd occasion		1st occasion	2nd occasion
	90th 95th 99th 10–90th	↓6.0% (ns) ↓17% (0.026) ↓11% (0.033) ↓7.9% (0.011)	↓34% (0.002) ↓29% (<0.001) ↓36% (<0.001) ↓31% (<0.001)	≥30° ≥45° ≥60°	↓13% (ns) ↓34% (0.015) ↓80% (0.026)	↓68% (0.001) ↓80% (<0.001) ↓89% (0.001)
	Directly after (≤8 h)					
	90th 95th 99th 10–90th	34% (0.002) ↓31% (0.001) ↓23% (0.003) ↓31% (<0.001)		≥30° ≥45° ≥60°	↓60% (<0.001) ↓61% (0.002) ↓67% (0.034)	
	Very short term (≤1 week)					
	90th 95th 99th 10–90th	↓12% (ns) ↓13% (ns) ↑1.7% (ns) ↓2.4% (ns)		≥30° ≥45° ≥60°	↓15% (ns) ↓3.4% (ns) ↓4.6% (ns)	
	Short term (≤1 month)					
	90th 95th 99th 10–90th	↓5.5% (ns) ↓10% (ns) ↓11% (ns) ↑0.1% (ns)		≥30° ≥45° ≥60°	↓7% (ns) ↓33% (ns) ↓44% (ns)	
Ribeiro et al. [74]	Group mean difference (compared to baseline) in frequency of episodes exceeding lumbar postural threshold ^1^					
	Control group			Intervention group		
	During feedback ^2^					
	↓0.3 times/h, ↓3.4% (ns)			↓0.6 times/h, ↓8% (ns)		
	Very short term (≤1 week)					
	↓0.6 times/h, ↓8% (ns)			↓0.6 times/h, ↓8% (ns)		
	Short term (≤1 month)					
	↓2.2 times/h, ↓30% (ns)			↓2.2 times/h, ↓30% (ns)		
	Midterm (<6 months)					
	↑0.4 times/h, ↑5.5% (ns)			↑3.3 times/h, ↑48.5% (ns)		
	Long term (≥6 months) ^3^					
	↑0.5 times/h, ↑6.2% (ns)			↓0.4 times/h, ↓5.91% (ns)		
	**Moderate quality studies**					
Bootsman et al. [83]	Group mean difference in frequency of poor posture (feedback condition vs. baseline)					
	During feedback administration					
	1st session: ↓3.5 times/min, ↓13.5% (sign ^4^)			2nd session^5^: ↓6.5 times/min, ↓25.3% (sign ^4^)		
	Directly after (≤8 h)					
	After 1st session: ↓0.7 times/min, ↓2.7% (ns)					

Notes: ns: not statistically significant; sign: statistically significant; ^1^ statistical test refers to the difference between the control group and the intervention group; ^2^ average of weeks 1–4; ^3^ averages of 6-month and 12-month follow-up; ^4^ significance level not reported; ^5^ Included is also visual feedback combined with note-taking.

**Table 13:** Summary of results from high-quality studies in controlled settings: Statistically significant decreases in exposures are indicated by green, non-statistically significant decreases by yellow, and non-statistically significant increases by orange, with *p*-values shown in parentheses.

Study	Reported Effects													
	Group mean (absolute) difference of max lumbosacral flexion (feedback group vs. control group)													
Boocock et al. [82]	During feedback													
			Flexion angle (°):		Lumbosacral ↓8% ^1,2^ (0.033) ^3^			Trunk ↓18.6% ^1,2^ (0.004) ^3^						
Kamachi et al. [84]	Group mean difference in distribution of lumbar spine flexion angle (intervention group vs. control group)													
	Care giving task							Skill transfer task						
	Directly after (≤8 h)													
	80th ↓17% (0.012)			95th ↓15% (0.036)										
	Short term (≤1 month)													
	80th ↓21% (0.001)			95th ↓23% (<0.001)				80th ↓ ^4^ (ns)			95th ↓ ^4^ (ns)			
	Midterm (≤6 months)													
	80th ↓14% (0.024)			95th ↓13% (0.024)				80th ↓ ^4^ (ns)			95th ↓ ^4^ (ns)			
Lim et al. [75]	Group mean difference in distribution of trunk flexion angle (feedback condition vs. baseline)													
	During feedback													
		Lifting-lowering task ^5^					Shoveling ^5^				Tying rebar ^5^			
		Back-position ^6^		Wrist-position ^7^			Back-position ^6^	Wrist-position ^7^			Back-position ^6^			Wrist-position ^7^
	50th 90th 95th	↓38% (<0.05) ↓18% (<0.05) ↓14% (<0.05)		↓48% (<0.05) ↓21% (<0.05) ↓15% (<0.05)			↓35% (<0.05) ↓15% (<0.05) ↓% ^4^ (ns)	↓34% (<0.05) ↓16% (<0.05) ↓15% (<0.05)			↓% ^4^ (ns) ↓% ^4^ (ns) ↓% ^4^ (ns)			↓% ^4^ (ns) ↑% ^4^ (ns) ↑% ^4^ (ns)
Lind et al. [73]	Median intra-individual differences in angle compared to baseline													
	Trunk inclination angle							Arm elevation angle						
	During feedback													
	Cumulative time			Distribution				Cumulative time				Distribution		
		1st	2nd		1st	2nd			1st	2nd			1st	2nd
	50th 90th 99th	↓23% (0.002) ↓31% (0.003) ↓37% (0.006)	↓31% (0.001) ↓31% (0.001) ↓37% (0.003)	≥20° ≥30° ≥45°	↓50% (0.003) ↓50% (0.004) ↓75% (0.007)	↓55% (0.001) ↓54% (0.002) ↓92% (0.007)		50th 90th 99th	↓5% (0.013) ↓7% (0.004) ↓3% (ns)	↓10% (0.006) ↓15% (0.002) ↓9% (ns)		≥20° ≥30° ≥45°	↓22% (ns) ↑3% (ns) ↑13% (ns)	↓30% (0.039) ↓11% (0.042) ↓4% (0.006)
	Directly after (≤4 h) ^8^													
	Cumulative time ≥20° ↓30% (0.001) ≥30° ↓35% (0.002) ≥45° ↓75% (0.005)			Distribution 50th ↓31% (0.001) 90th ↓12% (0.002) 99th ↓34% (0.003)				Cumulative time ≥20° ↓32% (0.033) ≥30° ↓19% (ns) ≥45° ↓4% (0.039)				Distribution 50th ↓10% (0.013) 90th ↓11% (0.004) 99th ↓7% (ns)		
Oppici et al. [87]	Median peak sacro-lumbar flexion angle of feedback group compared to controls (without feedback)													
	During feedback													
	↓5.8°, ↓27% (<0.01)													
	Directly after (≤8 h)													
	↓3.7°, ↓17% (<0.05)													

Notes: ns: not statistically significant; ^1^ absolute percentage of difference at the 20th minute compared to the 1st minute; ^2^ the angle of lumbosacral flexion and the trunk flexion increased for both groups at the 20th minute, but less for the feedback group in absolute; ^3^ calculated based on the slope of the trend from the 1st to 20th minute; ^4^ no values reported, only reported in a figure; ^5^ no statistically significant differences in exposure between the back-compared to wrist position; ^6^ vibration units positioned on the back; ^7^ vibration units on the wrist; ^8^ after the first training session.

**Table 14:** Summary of results from moderate-quality studies in controlled settings: Statistically significant decreases in exposures are indicated by green, non-statistically significant decreases by yellow, and non-statistically significant increases by orange, with *p*-values shown in parentheses.

Study	Reported Effects					
Agruss et al. [81]	Group mean difference in compression force (L5/S1) compared to baseline					
	Directly after (≤8 h)					
	Control group ↓280 N, ↓11% (ns ^1^)		Acc. Feedback group ↓680 N, ↓25% (ns ^1^; <0.01 ^2^)		EMG-feedback group ↓468 N, ↓17% (ns ^1^)	
Kernozek et al. [85]	Average maximum moment (Nm) compared to baseline					
	Short term (≤1 month)					
	Control group Side-bending: ↓0.5 Nm, ↓10% (NR) ^3^ Flexor: ↓1.2 Nm, ↓1% (NR) ^3^ Rotary: ↑0.2 Nm, ↑3% (NR) ^3^			Feedback group Side-bending: ↓4.4 Nm, ↓70% (NR) ^3^ Flexor: ↓44.2 Nm, ↓43% (NR) ^3^ Rotary: ↓0.7 Nm, ↓11 (NR) ^3^		
Langenskiöld et al. [76]	Group mean difference (feedback condition vs. baseline)					
	Trunk inclination angle			Arm elevation angle		
	Distribution		Proportion of the time	Distribution	Proportion of the time	
	During feedback					
	50th ↓41% (ns) 90th ↓12% (ns) 99th ↓9% (ns)		≥20° ↓15% (ns) ≥30° ↓19% (0.026) ≥45° ↓36% (0.008)	50th ↓9% (0.016) 90th ↓8% (0.003) 99th ↓3% (ns)	≥30° ↓11% (ns) ≥45° ↓18% (0.008) ≥60° ↓20% (0.002)	
	Directly after (≤8 h)					
	50th ↓94% (ns) 90th ↓18% (0.012) 99th ↓19% (0.008)		≥20° ↓23% (0.028) ≥30° ↓27% (0.014) ≥45° ↓54% (0.008)	50th ↓8% (0.002) 90th ↓8% (<0.001) 99th ↓5% (ns)	≥30° ↓10% (0.019) ≥45° ↓15% (0.002) ≥60° ↓17% (0.001)	
Lind et al. [86]	Group mean difference in arm elevation (feedback condition vs. baseline)					
	During feedback					
	Distribution	1st	2nd	Proportion of the time	1st	2nd
	50th 90th 95th 99th	↓32% (<0.001) ↓16% (<0.001) ↓10% (0.002) ↓13% (0.001)	↓33% (<0.001) ↓21% (0.001) ↓19% (0.001) ↓16% (<0.001)	≥30° ≥45° ≥60°	↓38% (<0.001) ↓36% (<0.001) ↓49% (0.001)	↓38% (<0.001) ↓45% (<0.001) ↓65% (<0.001)
Owlia et al. [72]	Difference in the distribution of lumbar spine flexion (feedback condition vs. baseline)					
	Directly after (≤8 h)					
	Distribution 50th 80th 95th		Control group ↓ ^4^ (ns) ↑ ^4^ (ns) ↑ ^4^ (ns)	Intervention group ↓ ^4^ (ns) ↓36% ^5^ (0.024 ^6^) ↓29% ^5^ (0.002)		
Punt et al. [70]	Difference against baseline (session 1)					
			Control group	Moment group	Inclination group	Lum. flex. group
	During feedback—(Session 2 and 3)					
	Peak moments L5/S1 (Nm)		↑ ^7^ (sign); ↑ ^7^ (sign)	↓ ^7^ (sign), ↓ ^7^ (sign)	↓ ^7^ (sign), ↓ ^7^ (sign)	↓ ^7^ (ns), → ^7^ (ns)
	Time above moment threshold (s)		↑ ^7^ (ns); ↑ ^7^ (ns)	↓ ^7^ (sign); ↓ ^7^ (sign)	↓ ^7^ (sign); ↓ ^7^ (sign)	↓ ^7^ (ns); ↓ ^7^ (ns)
	Trunk inclination angle (°)		↓ ^7^ (ns); ↓ ^7^ (ns)	↓ ^7^ (sign); ↓ ^7^ (sign)	↓ ^7^ (sign); ↓ ^7^ (sign)	↓ ^7^ (sign); ↓ ^7^ (sign)
	Trunk flexion angle (°)		↓ ^7^ (sign); ↓ ^7^ (ns)	↓ ^7^ (sign); ↓ ^7^ (sign)	↓ ^7^ (sign); ↓ ^7^ (sign)	↓ ^7^ (sign); ↓ ^7^ (sign)
	Directly after (≤8 h)					
	Peak moments L5/S1 (Nm)		↑Nm ^7^,↑6% (sign)	↓Nm ^7^, ↓9% (sign)	↓Nm ^7^, ↓11% (sign)	→ ^7^ (ns)
	Time above moment threshold (s)		↓ ^7^ (ns)	↓0.24 s ^7^ (sign)	↓0.26 s ^7^ (sign)	↓0.1 s ^7^ (ns)
	Trunk inclination angle (°)		↓ ^7^ (ns)	↓20° ^7^ (sign)	↓25° ^7^ (sign)	↓25° ^7^ (sign)
	Trunk flexion angle (°)		↓4° ^7^ (ns)	↓14° ^7^ (sign)	↓19° ^7^ (sign)	↓16° ^7^ (sign)

Notes: NR, nor reported; ns, not statistically significant; sign, not statistically significant;^1^ Interaction between the improvement and the group (*p* = 0.023); ^2^ The comparative improvement between the control group and the group receiving verbal acceleration feedback (*p* < 0.01); ^3^ differences were statistically significant for the pooled group (feedback group and control group); no separate statistical test for each group was reported. ^4^ no values reported, value estimated from figure); ^5^ a fraction of the reduction may be attributed to the video training; ^6^ derived from Lind [67] since reported (incorrectly) as 0.24 in the article by Owlia et al. [72]; ^7^ values reported only in figures.

**Table 15:** Summary of the consistency of the evidence for the effectiveness of sensor-based augmented feedback on manual handling in real work environments and controlled settings.

Study	During Feedback	Directly After (≤8 h)	Very Short Term (≤1 Week)	Short Term (≤1 Month)	Midterm (≤6 Months)	Long Term (≥6 Months)
**Real Work Environments**						
Strength of Evidence	Inconsistent	Very limited	No evidence	No evidence	No evidence	No evidence
**High quality studies**						
Lind et al. [77]	++	++	(+)/=	(+)/=		
Ribeiro et al. [74]	=		=	=	=	=
**Moderate quality studies**						
Bootsman et al. [83]	++	(+)/=				
**Controlled settings**						
Strength of Evidence	Strong	Strong ^4^		Limited ^1^/No evidence ^2^	Limited ^1^/No evidence ^2^	
**High quality studies**						
Boocock et al. [82]	++					
Kamachi et al. [84]		++		++ ^1^/= ^2^	++ ^1^/= ^2^	
Lim et al. [75]	+					
Lind et al. [73]	++	++				
Oppici et al. [87]	++	++				
**Moderate quality studies**						
Agruss et al. [81]		+/=				
Kernozek et al. [85]				*		
Langenskiöld et al. [76]	+	++				
Lind et al. [86]	++					
Owlia et al. [72]		++ ^3^/= ^4^				
Punt et al. [70]	++/+	++/+				

Notes: ++ overall, consistent statistically significant findings indicate that feedback reduces biomechanical exposure (dark green); + overall, statistically significant findings indicate that feedback reduces biomechanical exposure (light green); +/= mixed results with some significant findings indicating that feedback reduces biomechanical exposure and insignificant results in one or both directions (yellow); (+)/= overall, insignificant results of a tendency indicating that feedback reduces biomechanical exposure (light orange); = overall, insignificant results in both directions (light orange); * the results are unclear due to an incomplete report of the statistical analysis; ^1^ effect based on task included in feedback training; ^2^ effect based on task not included in feedback training; ^4^ peak exposure.

## Data Availability

Not applicable. Database searches were conducted only for this review.

## **Appendix A.** Search Strings in the Literature Search

**Table A1:** The search strings used in Medline on 9 June 2024.

“feedback” OR “biofeedback” AND “posture$” OR “postural” OR “movement$” OR “muscle activity” OR “EMG” AND “neck” OR “trunk” OR “spine” OR “back” OR “arm$” or “wrist$” OR “hand$” The following terms were excluded “gait”, “child*”, “rehab*”, “parkinson”, “stroke*”, “cerebral palsy”, “spinal cord injury”, “prosthesis”, and “therap*”. The first two search strings were applied “all fields”, while the third as well the exclusion were applied to the abstract. Articles in other languages than English were excluded. The search covered the period 1 January 2020 to 9 June 2024.

**Table A2:** The search strings used in Web of Science on 9 June 2024.

“feedback” OR “biofeedback” AND “posture$” OR “postural” OR “movement$” OR “muscle activity” OR “EMG” AND “neck” OR “trunk” OR “spine” OR “upper back” OR “lower back” OR “arm$” or “wrist$” OR “hand$” The following terms were excluded: “gait”, “child”, “rehab”, “parkinson”, “stroke*”, “cerebral palsy”, “spinal cord injury”, “prosthesis”, and “therap*”. The search terms were applied to the abstract. Review articles were excluded. Articles in other languages than English were excluded. The search covered the period 1 January 2020 to 9 June 2024.

## **Appendix B.** The Criteria to Assess the Methodological Quality

**Table A3:** Criteria for assessing the methodological quality of observational cohort and cross-sectional studies.

Item	Criteria
1. Clear research question	Clearly stated research question or study objective, including a description of the outcome (dependent variable)
2. Clear study population	Clearly defined study population and inclusion/exclusion criteria, and consistently applied inclusion/exclusion criteria
3. Participation rate	Participation rate ≥50% of eligible persons of the identified pool of eligible persons
4. Subjects recruitment	Subjects recruited from the same/similar populations, with uniformly applied inclusion/exclusion criteria
5. Justified sample size	Sample size sufficiently justified and large enough to detect a difference in the outcome with at least 80% power
6. Temporality of exposure(s) and outcome(s)	Exposure(s) measured before outcome(s)
7. Sufficient time	Timeframe for feedback described and sufficient time to induce behavioral changes
8. Dependent variable scaling	Dependent variable (e.g., posture, movements, or muscle activity) assessed on a continuous or categorical scale
9. Independent variables treatment	Relevant independent variables controlled or measured (as minimum, the amount of work performed per time unit, and a clearly defined and consistently implemented feedback trigger)
10. Assessment of dependent variable	Dependent variable(s) assessed more than one time
11. Assessment of dependent variable	Dependent variable clearly defined and adequately assessed
12. Blinding	Assessors blinded to the participants’ group allocation
13. Loss to follow-up	Data from at least 80% of participants were included in the final analysis
14. Control of confounders	Key confounding variables that could alter the outcome results were adjusted for

**Table A4:** Criteria for assessing the methodological quality of controlled intervention studies.

Item	Criteria
1. Study description	This study is described as a randomized controlled trial and provides adequate details of the study design
2. Randomization	Suitable randomization was employed, such as computer-generated random allocation of participants
3. Concealed allocation	Concealed process of assigning participants to group allocation
4. Blinding (providers and participants)	Blinding (group assignment) of both the administrator of the intervention and the participants receiving the intervention
5. Blinding (assessors)	Blinding of assessors evaluating outcomes to participants’ group allocation
6. Baseline characteristics	Baseline characteristics reported and balanced between groups, such as age, gender, experience, occupation, job exposure, and disorders
7. Endpoint dropout	The dropout rate ≤20% at the end of the study for each group
8. Endpoint dropout (between groups)	Dropout rate ≤15% between groups at the end of the study
9. Adherence	Adherence to the intervention protocol by participants in all group
10. Confounding interventions	No additional interventions that could confound the study results were occurring, or any such interventions were similar between groups
11. Quality of outcomes assessment	Dependent variable(s) measured using accurate and precise tools/methods
12. Justified sample size	Sample size sufficiently justified and large enough to detect differences in the outcome between groups with at least 80% power
13. Prespecified analysis	Predetermined analysis of the outcomes
14. Group assignment	Participants were analyzed based on their original group allocation
